# Characterization of metabolic features derived from the non-polar metabolite UHPLC-QTOF dataset of *Jaspis sp.* Collected from the waters off Pulau Banggi, Sabah, Malaysia

**DOI:** 10.1016/j.dib.2025.111474

**Published:** 2025-03-22

**Authors:** Dexter Jiunn Herng Lee, Yee-Soon Ling, Christopher Lok Yung Voo, Mok Sam Lum, Jualang Azlan Gansau

**Affiliations:** a Biotechnology Research Institute, Jalan UMS, Universiti Malaysia Sabah, Kota Kinabalu, Sabah 88400, Malaysia; b Inanam, Off Jalan Tuaran, Alpha Frontier HSE Consultancy Sdn Bhd, Unit No. 3 & 4, 21 Jalan Burung Keleto, Kota Kinabalu, Sabah 88450, Malaysia; c Not affiliated; d Faculty of Sustainable Agriculture, Universiti Malaysia Sabah, Locked Bag No. 3, Sandakan, Sabah 90509, Malaysia; e Faculty of Science and Natural Resources, Jalan UMS, Universiti Malaysia Sabah, Kota Kinabalu, Sabah 88400, Malaysia

**Keywords:** Marine sponge, *Jaspis sp*, Untargeted metabolomics, UHPLC-QTOF, Environmental sample

## Abstract

The marine biome is a rich source of bioactive compounds. The discovery of anti-cancer compounds in *Cryptotheca crypta* in 1950 initiated a wave of bioprospecting efforts focused on marine sponges. *Jaspis* sp., a marine sponge, has been reported to exhibit anti-cancer activity against human colorectal cancer. A sample of *Jaspis* sp., collected from the waters off Pulau Banggi, Sabah, Malaysia, was analyzed through a non-polar metabolite survey. The non-polar crude extract was profiled using UHPLC-QTOF in both positive and negative modes. The detected metabolic features were clustered, and representative features were tentatively identified through a combination of spectral database searches (using various MS2 spectral databases) and *in silico* compound identification. This dataset provides a valuable foundation for future bioprospecting endeavors involving *Jaspis* sp.

Specifications Table

**Table:** 

Subject	Chemistry.
Specific subject area	Natural product research, Mass spectrometry.
Type of data	Table, Figures Raw, Analyzed.
Data collection	The MS2 data of the non-polar metabolites from *Jaspis sp.* was obtained using ultra high-performance liquid chromatography (Thermo Fischer Scientific's Vanquish^TM^ Horizon UHPLC system) high resolution tandem mass spectrometry (Bruker Corporation's Impact II Q-TOF mass spectrometer).
Data source location	The *Jaspis sp.* sample was obtained from a previous study conducted by Yong *et. al.* [1], which in turn was collected from the waters off Pulau Banggi, Sabah, Malaysia (7°04’51.0”N, 117°03’12.0”E) on 15 June 2016 through scuba diving and was subsequently morphologically identified as *Jaspis sp.* with the specimen label KDT18. The specimen was relabelled as SP18 in this study*.*
Data accessibility	Repository name: Mendeley Repository Data identification number: http://dx.doi.org/10.17632/p4zt8vfdjv.2 [2] Direct URL to data: http://dx.doi.org/10.17632/p4zt8vfdjv.2 Instructions for accessing these data: …
Related research article	None*.*

## 1 Value of the Data

• This dataset shows the detected metabolic features (identified or otherwise) found in *Jaspis sp.* isolated off the waters of Pulau Banggi (Banggi Island), Sabah, Malaysia.
• Marine biome is a source of bioactive compounds and members of the Jaspis genus had been shown to possess bioactive compounds with numerous useful effects, such as antimicrobial [3] and anti-cancer [1] activities.
• This study takes an untargeted approach in identifying the non-polar metabolic features detected in the non-polar metabolite survey of *Jaspis sp.* sample isolated from Pulau Banggi. The tentative identifications can serve as a starting point for any future bioprospecting works pertaining to *Jaspis sp.* in general.

## 2 Background

The marine biome is rich source of bioactive compounds, some of which are potentially novel. The discovery of bioactive compounds with anti-cancer activity in *Cryptotheca crypta* in 1950 [4] kicked off a slew of bioprospecting works targeting marine sponges. A previous study on *Jaspis sp.* isolated from the waters off Pulau Banggi, Sabah, Malaysia has shown that its crude extract exhibited anti-cancer properties against colorectal cancer cells [1]. A metabolite survey of the same sample would provide insights into other potentially bioactive compounds present in the said crude extract and serve as a starting point for future bioprospecting works.

## 3 Data Description

**Fig. 1:**
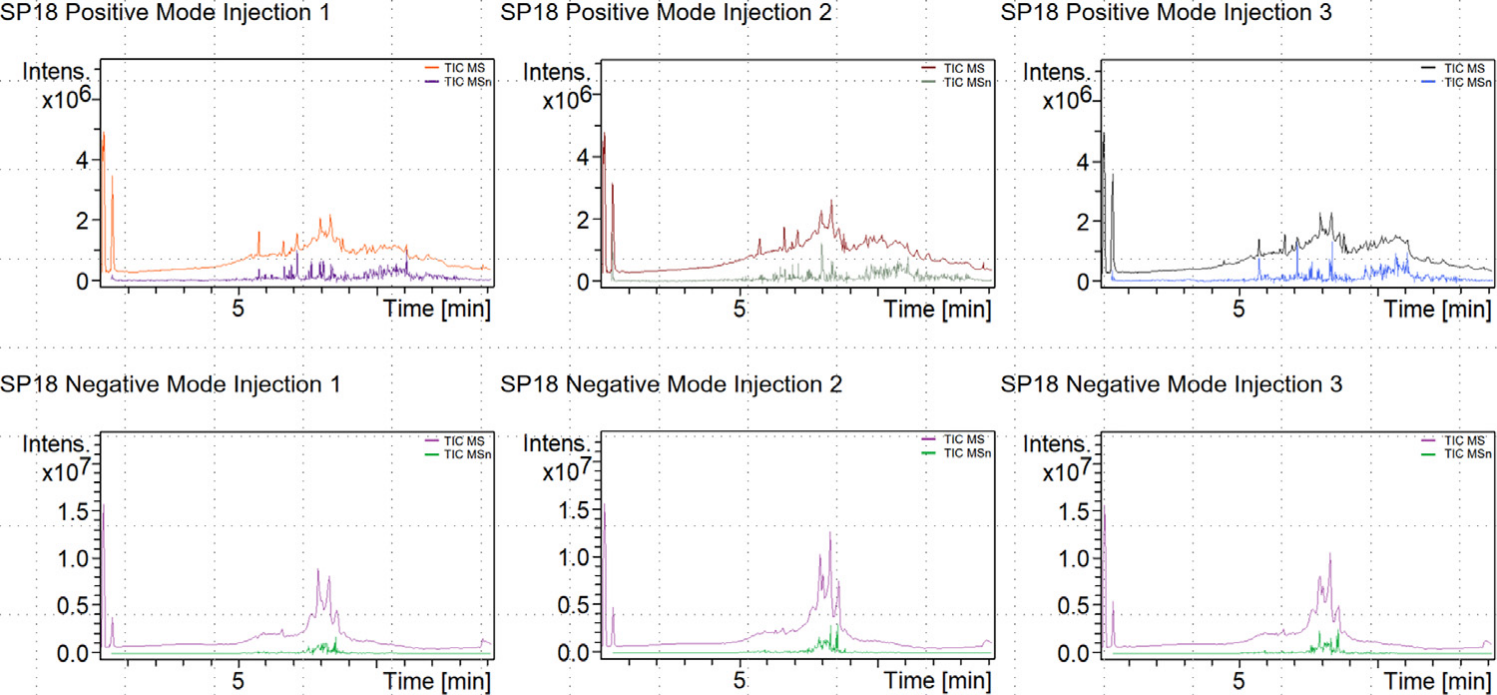
Total ion chromatograms for the sample injections in both positive and negative modes with 3 injections for each mode.

Fig. 1 shows the total ion chromatograms (MS and MSn) for sample injections. Table 1 shows the tentatively identified non-polar metabolic features found in the *Jaspis sp.* crude extract sample, with emphasis on the identification method, injection, mode, detected m/z, detected retention time, compound name, formula, InChIKey, class and ChEBI types and roles. The raw data can be accessed through the Mendeley data repository at http://dx.doi.org/10.17632/p4zt8vfdjv.1 under Appendix 1.

**Table 1:** Multistep gradient setting use for the UHPLC system.

Time (minutes)	Solvent A (%)	Solvent B (%)
0.0	99	1
0.5	99	1
5.0	40	60
10.0	0	100
13.0	0	100
13.1	99	1
14.0	99	1

## 4 Experimental Design, Materials and Methods

### 4.1 Sample Collection and Non-Polar Metabolite Extraction

The frozen (-40 °C) *Jaspis sp.* sample obtained from a previous study conducted by Yong et. al. [1] was freeze dried, and subsequently homogenized using a handheld homogenizer. Twelve mg of the homogenized sample were used for non-polar metabolite liquid-liquid extraction using Folch's extraction buffer [5] with modifications. The weighted homogenized sample was added into a glass test tube, followed by HPLC grade chloroform (Merck), HPLC grade methanol (Merck), and water (obtained from Mili-Q water purification system) in a 1:1:1 ratio (total volume of 15 mL) using a glass pipette. The mixture in the glass tube was thoroughly mixed using a vortex shaker for 10 s. Phase separation was done by centrifuging the mixture in the glass test tube at 250 g at 4 °C for 15 min using a refrigerated centrifuge. The non-polar phase (chloroform layer; lower layer) was carefully transferred into 2 smaller (4 mL) glass test tubes. The chloroform was evaporated by using a vacuum concentrator (Eppendorf Concentrator Plus) at 30 °C and V-AQ mode with centrifugation. The desiccated non-polar metabolites were resuspended using 2 mL of HPLC grade methanol (Merck) for each of the 4 mL glass test tubes. Due to the issue highlighted by Puah et. al. with regards to the extractable impurities found in fluoropolymer-based membrane filters interfering with high-throughput untargeted metabolomics analysis [6], the resuspended non-polar metabolites were subjected to centrifugation in the same vacuum concentrator for 30 min at ambient temperature with the vacuum pump switched off. The resulting supernatant was carefully (without disturbing the sediment) transferred into a 2 mL screw top glass vial, capped, and stored at -20 °C.

### 4.2 MS2 Profiling Using UHPLC-HRMSMS

Reversed-phase UHPLC-HRMSMS was used to profile the extracted non-polar metabolites. The UHPLC-HRMSMS setup consists of a Thermo Fischer Scientific's Vanquish^TM^ Horizon UHPLC system coupled to a Bruker Corporation's Impact II Q-TOF mass spectrometer. The reversed-phase liquid chromatography uses a multistep gradient with a binary solvent set up, with Solvent A consisting of MS-grade water (Merck), 1 % (v/v) 1 M ammonium acetate (Merck) and 0.1 % (v/v) formic acid (HPLC grade, Merck), while Solvent B consists of an acetonitrile (HPLC-grade, Merck)-methanol (HPLC-grade, Merck) mixture (3: 2 ratio), 1 % (v/v) 1 M ammonium acetate (Merck) and 0.1 % (v/v) formic acid (HPLC grade, Merck). The multistep gradient was set as per Table 1. The flow rate was set to 0.5 mL/min while the pressure was set at 1000 bar. The column oven was set and maintained at 40 °C throughout the duration of the entire gradient. The column used was the Phenomenex Kinetex F5 pentafluorophenyl column (2.1 × 100 mm, 1.7 µm), coupled together with the Phenomenex SecurityGuard ULTRA cartridges UHPLC F5 column guard. The Q-TOF mass spectrometer was operated with the parameters as listed in Table 2. The MS2 data was acquired in both positive and negative modes for the sample, blanks (HPLC-grade methanol), as well as non-polar phase of the extraction buffer (i.e. extraction buffer without the sample). Eighty-seven technical replicates (multiple injections on the same sample) were done on the blanks (43 in positive mode, 44 in negative mode), 6 technical replicates on the sample (3 in positive mode, 3 in negative mode), and 6 technical replicates on the non-polar phase of the extraction buffer (3 in positive mode, 3 in negative mode).

**Table 2:** Parameters used for Impact II Q-TOF mass spectrometer.

Parameters	Positive Mode	Negative Mode
**Source**		
Source Type	ESI	
Focus	Not active	
Scan Begin	50 m/z	50 m/z
Scan End	1500 m/z	1200 m/z
Ion Polarity	Positive	Negative
Set Capillary	4500 V	
Set End Plate Offset	-500 V	
Set Charging Voltage	2000 V	
Set Corona	0 nA	
Set Nebulizer	3.0 Bar	
Set Dry Heater	250 °C	
Set Dry Gas	12.0 l/min	
Set Divert Valve	Waste	
Set APCI Heater	0 °C	
**Ion Optics**		
Set Funnel 1 RF	300.0 Vpp	
Set Funnel 2 RF	300.0 Vpp	
isCID Energy	0.0 eV	
Set Focus 1 - Lens 2	34.3 V	-32.5 V
Set Focus 1 - Lens 3	-42.8 V	54.7 V
Set Focus 2 - Lens 1	35.0 V	-35.0 V
Set Focus 2 - Lens 2	-26.3 V	30.9 V
Set Focus 2 - Lens 3	29.0 V	-28.5 V
Set Storage	40.0 V	-40.0 V
Set Extraction	23.0 V	-21.0 V
Set Focus 3 - Lens 2	0.0 V	0.0 V
Set Focus 3 - Lens 3	-72.0 V	-24.7 V
Set Focus 3 - Lens 4	0.0 V	3.3 V
Set Focus 3 - Lens 5	-0.0 V	-19.0 V
**Quadrupole**		
Set Ion Energy (MS Only)	5.0 eV	-5.0 eV
Set Isolation Mass (MS Only)	100.0 m/z	100.0 m/z
Collision Energy	10.0 eV	-10.0 eV
Set Collision Cell RF	800.0 Vpp	600.0 Vpp
Set Transfer Time	82.5 µs	70.7 µs
Set PrePulseStrorage Time	2.0 µs	5.0 µs
**Mass Calibration**		
TOF Calibration Version	Version 1	Version 2
TOF1 Calibration Mode	Quadratic + HPC	
TOF2 Calibration Mode	-1	Enhanced Quadratic
**TOF**		
Set Corrector Fill	73.0 V	82.2 V
Set Corrector Extract	700.0 V	300.0 V
Set Corrector Lens	6280.0 V	6577.0 V
Set Reflector	2600.0 V	2780.0 V
Set Decelerator	765.0 V	624.0 V
Set Flight Tube	9900.0 V	
Set Pulser Push	1600.0 V	
Set Detector TOF	2225.0 V	
**Processing**		
Summation	9642 x	9939 x
Guessed Noise	200	
Peak Width	5 pts	
Average Noise	10	
Guessed Average	100	

### 4.3 Data Acquisition, Processing, and Reporting

The data obtained was processed using Bruker Corporation's Compass DataAnalysis 4.3 (Build 110.102.1532) software, where the metabolic features were detected using AutoMS(n), with the intensity threshold set at 100 for both positive and negative modes, retention time window unchecked, and the background subtraction set to spectral, while the “Maximum charge” setting for “Charge Deconvolution” was set to “Auto”. The detected metabolic features for each injection were exported in MGF format (one file per injection), after which the MGF files were converted into MSP formatted files. NIST MSPepSearch (version 0.96) was used using the standard MSMS search mode (settings as per Table 3) to remove metabolic features detected in blank and non-polar extraction buffer injections that match the metabolic features detected in the sample injections with a minimum match factor of 800. The remaining metabolic features from all sample injections were consolidated via spectral matching using MSPepSearch, in which metabolic features with a minimum match factor of 800 were clustered together and the metabolic feature with the highest intensity was chosen for each of the clusters formed. metabolic features which are unable to match themselves (i.e. self-matching) were removed. Identification of the remaining metabolic features was done using a combination of spectral database search and *in silico* compound identification. Spectral database search was done using MSPepSearch in both standard MSMS and Hybrid MSMS modes (settings as per Table 3) using NIST20 libraries, MoNA [7] LC-MS/MS spectral libraries, Lipidblast [8] MSMS spectral library, and MS-DIAL [9] MSMS libraries, with matches having a minimum match factor of 800 being kept as recommended by Kind *et. al.* for good matches (match factor 850) [10] with a slight leeway. *In silico* compound identification was done using Sirius (version 5.8.3) [[11], [12], [13], [14], [15], [16]] (settings as per Table 4) using the built-in databases as well as a custom database consisting of compounds from CMNPD [17] Porifera organisms, Lipidblast, MS-DIAL, MoNA, and NIST20 databases. The identification results were consolidated, and in cases where a metabolic feature was identified in more than one identification method, the identification is ranked based on the search method used with priority given to standard MSMS search identification (sorted by descending match factor), followed by Hybrid MSMS search identification (sorted by descending match factor), and finally Sirius *in silico* compound identification. The highest ranked identification was selected while the others were discarded. Information regarding the identified compound's pathway, superclass, class, and subclass was obtained using NPClassifier [18] and Classyfire [14], while the compound's application, biological role, and chemical role (where applicable) was obtained by searching through ChEBI [19]. Chemspider [20] was used to obtain the identified compound's SMILES, which is needed for NPClassifier.

**Table 3:** NIST MSPepSearch settings for standard MSMS search and Hybrid MSMS search modes.

Parameter	Option/Flag	Value	Remarks
HiRes Search	P	-	-
HiRes Search Type	z	-	Standard MSMS search mode
	y		Hybrid MSMS search mode
Presearch type	d	-	-
Peptide HiRes search (P) scoring options	q	-	-
HiRes search option	z	-	-
HiRes search option	i	-	-
Search parameters	/Z	1.6	-
Search parameters	/M	0.8	-

**Table 4:** Settings used for Sirius *in silico* compound identification.

Main Options	Sub-options	Positive	Negative
SIRIUS - Molecular Formula Identification	Use DB formulas only	All	
	Possible ionizations	[M+H]+, [M+Na]+	All
CSI:FingerID - Fingerprint Prediction	Fallback Adducts	[M + H]+, [M + H3N + H]+, [M + Na]+	[M + Br]-, [M + Cl]-, [M - H], [M + CH2O2 - H]-, [M + C2H3N – H]-
CSI:FingerID - Structure Database Search	Search DBs	All	

## Limitations

Some of the detected metabolic features may originate from other organisms (e.g. symbiotes) and pollutants present together with the *Jaspis sp.* sample at the time of the sample collection*.*

## Ethics Statement

This research work does not require ethical approval.

## CRediT authorship contribution statement

**Dexter Jiunn Herng Lee:** Data curation, Formal analysis, Investigation, Methodology, Visualization, Writing – original draft. **Yee-Soon Ling:** Conceptualization, Funding acquisition, Resources, Project administration, Supervision, Writing – review & editing. **Christopher Lok Yung Voo:** Supervision, Writing – review & editing. **Mok Sam Lum:** Supervision, Writing – review & editing. **Jualang Azlan Gansau:** Supervision, Writing – review & editing.

## Data Availability

Mendeley DataResearch data (Original data).

## Appendix Supplementary materials

Image, application 1
